# Development and validation of a predictive model for pathogenic *Escherichia coli* in fresh‐cut produce

**DOI:** 10.1002/fsn3.2642

**Published:** 2021-10-29

**Authors:** You Jin Kim, Ju Yeon Park, Soo Hwan Suh, Mi‐Gyeong Kim, Hyo‐Sun Kwak, Soon Han Kim, Eun Jeong Heo

**Affiliations:** ^1^ Food Microbiology Division Food Safety Evaluation Department Ministry of Food and Drug Safety Cheongju South Korea

**Keywords:** Baranyi model, food poisoning, growth model, lag phase duration, microbial contamination

## Abstract

This study was performed to develop and validate a predictive growth model of pathogenic *Escherichia coli* to ensure the safety of fresh‐cut produce. Samples were inoculated with a cocktail of seven *E. coli* strains of five pathotypes (EHEC, Enterohemorrhagic *E. coli*; ETEC, Enterotoxigenic *E. coli*; EPEC, Enteropathogenic *E. coli*; EIEC, Enteroinvasive *E. coli,* and EAEC, Enteroaggregative *E. coli*) and stored at 4, 10, 12, 15, 25, 30, and 37°C. Growth of pathogenic *E. coli* was observed above 12°C. The primary growth model for pathogenic *E. coli* in fresh‐cut produce was developed based on the Baranyi model. The secondary model was developed as a function of temperature for lag phase duration (LPD) and maximum specific growth rate (μ_max_) based on the polynomial second‐order model. The primary and secondary models for pathogenic *E. coli* were fitted with a high degree of goodness of fit (R^2^ ≥ 0.99). The bias factor (B_f_), accuracy factor (A_f_), and root mean square error (RMSE) were 0.995, 1.011, and 0.084, respectively. The growth model we developed can provide useful data for assessing the quantitative microbial risk of pathogenic *E. coli* in fresh‐cut produce intended for human consumption. In addition, it is thought to be widely available in industries that produce, process, distribute, and sell fresh‐cut produce.

## Graphical abstract

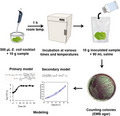

## 1 INTRODUCTION

Consumption of fresh‐cut produce has increased in recent years due to changes in the dietary patterns of consumers and their growing interest in health; fresh‐cut produce has been sold in various forms with vegetables and fruits being the main ingredients (Jo et al., 2011). According to the Food Sanitation Act, fresh‐cut produce is defined as agricultural or forest products that have undergone processing operations, such as washing, peeling, chopping, or shredding, or products with the simple addition of food or food additives, such as salads or sprout vegetables, that can be consumed as such (MFDS, 2020). Domestic sales of fresh‐cut produce reached 181.7 billion KRW in 2018, up to 7.9‐fold in total from 10 years before, growing at an annual rate of 22.9% from 2008. In particular, sales have been strong recently, with an enormous 48.3% increase between 2016 and 2017 (Kim et al., 2019). However, as most fresh‐cut produce are raw and consumed directly without undergoing separate cooking processes, they can be easily exposed to bacteria that can cause food poisoning; the number of food poisoning cases derived from fruits and vegetables has been increasing worldwide (Jo et al., 2011). According to food poisoning statistics in Korea, 41.8% of cases with known causes occurred between 2012 and 2016 and were found attributable to vegetables, a higher proportion than those caused by meat or fish (MFDS, 2019). In Spain, Garcia‐Gimeno et al. (1996) reported that out of 70 mixed vegetable samples, 21 contained *Listeria monocytogenes*, and in Korea, Kim et al. (2004) reported that microbes such as *Escherichia coli*, *Salmonella* spp., and *Staphylococcus aureus*, were detected in salads. In the United States, 16 people died from eating melons contaminated with *Listeria* (Lomonaco et al., 2013), and in Germany, 46 people died owing to food poisoning caused by organic vegetables contaminated with *E*. *coli* O104:H4 (Frank et al., 2011). Furthermore, in the United States, 43 people in 12 states fell sick after consuming romaine lettuce contaminated with enterohemorrhagic *E*. *coli* (EHEC) ahead of thanksgiving in 2018, including one person who developed hemolytic uremic syndrome, which prompted the Centers for Disease Control and Prevention (CDC) to recommend that romaine lettuce should not be consumed (CDC, 2018). According to the Ministry of Food and Drug Safety (MFDS), pathogenic *E*. *coli* is the second most common cause of food poisoning in Korea following norovirus (MFDS, 2019). *E*. *coli* are gram‐negative bacilli, which are part of the normal flora in humans and warm‐blooded animals and are mostly harmless to the human body; however, some of them have pathogenicity. On that basis, *E. coli* infections are divided into six pathotypes: Enterohemorrhagic *E. coli* (EHEC), Enterotoxigenic *E. coli* (ETEC), Enteropathogenic *E. coli* (EPEC), Enteroinvasive *E. coli (EIEC),* Enteroaggregative *E. coli (*EAEC), and Diffusely Adherent *E. coli* (DAEC) (Clements et al., 2012). *E. coli* strains can be categorized by serogroup, for example, *E. coli* O157, which causes severe infectious diarrhea or acute enteritis. Ingesting food contaminated with pathogenic *E*. *coli* can cause food poisoning, which, in severe cases, can result in death (Hwang et al., 2019; Yun et al., 2013).

Studies in the area of predictive food microbiology, as a new technology for securing safety in relation to food sanitation, have been actively conducted. These studies can predict changes in pathogenic microorganisms by mathematically describing changes in characteristics such as growth and death of microorganisms in food under certain environmental conditions (Whiting, 1995). Predictive models have been recognized as a means of predicting the growth of microorganisms in food, depending on internal factors, such as pH and water activity, and external factors, including temperature and time (McDonald & Sun, 1999). Predictive models have been considered highly effective in preventing food poisoning, and currently, predictive growth models are being developed in several countries including Korea for various pathogenic microorganisms that exist in raw ingredients and ready‐to‐eat foods (Park et al., 2007, 2010). In Korea, Cho et al. characterized pathogenic types of 111 *E. coli* isolates from diarrheal patients and five pathotypes, most prevalent in Korea, were evaluated with 49 STEC, 20 EPEC, 20 ETEC, 20 EAEC, and 2 EIEC isolates (Cho et al., 2010).

Therefore, the aim of this study was to develop a predictive growth model of *E*. *coli* in fresh‐cut produce for the most frequently emerged pathotypes in Korea, to ensure food safety, such as the prevention of food poisoning, and to use it for quantitative microbial risk assessment.

## 2 MATERIALS AND METHODS

### 2.1 Purchase of samples

Fresh‐cut produce (consisting of lettuce and cabbage) with the same lot number was purchased at a supermarket located in Sejong City, placed in a cooler, and immediately used in experiments after being transported to the laboratory within an hour. As the first step, the absence of *E. coli* in each purchased sample was confirmed according to Korea Food Code microbiological method (MFDS, 2020). Samples of the fresh‐cut produce were collected in aseptic conditions, with 10 g each in sterilized containers.

### 2.2 Measurement of pH and water activity

As major factors affecting the growth and development of *E. coli,* pH and water activity were measured prior to the development of the predictive growth model. pH was measured using a pH meter (Thermo Fisher Scientific, Waltham, Massachusetts, USA) after homogenization by mixing 90 ml of deionized distilled water with 10 g of sample. Water activity was measured using a water activity meter (Novasina, Lachen, Schwyz, Switzerland) after filling the sample cup (40 mm diameter × 12 mm deep) to approximately two‐thirds with the sample chopped into small pieces.

### 2.3 Bacterial strains and inoculation of the bacterial solution

Seven *E. coli* strains—*E*. *coli* NCCP 13,720(EHEC), 11,213(EHEC), 11,076(EHEC), 15,663(EIEC), 13,713(EPEC), 15,732(ETEC), and 14,039(EAEC)—used in this study were kept frozen at −80°C. Each bacterium was inoculated into tryptic soy broth (TSB, Oxoid, Basingstoke, UK) and simultaneously incubated at 37°C for 24 h. Our preliminary observation clarifies that there were no significant differences (*p* < .05, data not shown) in growth rates for the seven *E. coli* strains when they were cultured separately. Therefore, each 1 ml of seven cultured *E. coli* strains was mixed as a cocktail, centrifuged (2500 g, 4°C, 10 min), and washed twice in sterile peptone water (0.1%). The final cell pellets (approximately 7–8 log CFU/g) were resuspended in 10 ml of sterile peptone water (0.1%) for the inoculation. Then, 100 µl of this suspension was diluted in 400 µl of 0.1% sterile peptone water, which was spot‐inoculated evenly onto each of the 10 g fresh‐cut produce samples to make the final concentration of 4 log CFU/g. These *E. coli*‐inoculated samples were dried on a clean bench at room temperature for 1 h to ensure that seven *E. coli* cocktail strains were well attached to the surface of the salad samples. Considering the temperatures at the processing and distribution stages, inoculated samples were stored at 4, 10, 12, 15, 25, 30, and 37°C, and changes in bacterial counts were examined at each specific time period. In order to confirm that the initial inoculation was successful, the uncultured control (0 h) was used to calculate the concentration.

### 2.4 Bacterial cell counts

Samples of incubated fresh‐cut produce were taken out at each specific time period and placed in a sterilized bag, to which 90 ml of 0.85% saline solution was added, and homogenized for 1 min using a Stomacher (Interscience, Saint‐Nom‐la‐Bretèche, France). Subsequently, 1 ml of each homogenate was serially diluted (10‐fold) with saline, and 100 µl of each serial dilution was spread onto eosin methylene blue agar plates (EMB, Oxoid, Basingstoke, UK) and incubated at 37°C for 24–48 h. Thereafter, bacterial cell counts were determined by counting colonies with a metallic green sheen.

### 2.5 Development of the primary predictive growth model

To develop the first model, the lag phase duration (LPD) and maximum specific growth rate (μ_max_), which are the growth parameters of pathogenic *E*. *coli*, were calculated according to incubation temperatures. The Baranyi model (Baranyi & Roberts, 1994), using the DMfit program, was applied to the calculations of LPD and μ_max_. The Baranyi formula is as follows:

(1) $$N_{t}=N_{0}+\mu_{\max}\times A_{t}‐\ln\left[1+\frac{\exp\left(\mu_{\max}\times A_{t}\right)‐1}{\exp\left(N_{\max}‐N_{0}\right)}\right]$$

(2) $$A_{t}=t+\frac{1}{\mu_{\max}}\ln\left(\frac{\exp\left(‐\mu_{\max}\right)+q_{0}}{1+q_{0}}\right)$$

‐ A_t_: adjustment function
‐ μ_max_: maximum specific growth rate
‐ N_0_: initial bacterial cell counts
‐ N_max_: final bacterial counts
‐ q_0_: a parameter defining the initial physiological state of cells
‐ t: time

The coefficient of determination (R^2^) provided by DMfit was used to assess the data fitness.

### 2.6 Development of the secondary predictive growth model

To predict any changes induced by temperature on the LPD and the μ_max_ obtained through the primary predictive growth model, the secondary predictive growth model was developed using the polynomial second‐order model (McKeekin et al., 1993). The formula is as follows:

(3) $$\mathrm{LPD}\;\mathrm{or}\;\mu_{\max}=a+\left(b\times T\right)+\left(c\times T^{2}\right)$$

‐ a, b, c: constant
‐ T: temperature

### 2.7 Validation of developed predictive growth model

To verify the fitness of the developed predictive growth model formula for pathogenic *E. coli*, statistical indicators such as root mean square error (RMSE), bias factor (B_f_), and accuracy factor (A_f_) were calculated with fresh‐cut produce stored at 20°C, a temperature not used in the development of the model. RMSE is a figure determined using the difference between observed values and predicted ones, where the closer the value is to zero, the better the fitness of the developed predictive model (Baranyi et al., 1996).

(4) $$\mathrm{RMSE}=\sqrt{\frac{{\sum (pred\;‐\;obs)}^{2}}{n}}$$

‐ pred: predicted value
‐ obs: observed value
‐ n: number of observations

B_f_ is an assessment of the difference between observed values, determined by experiments, and mean values induced from the secondary model formula. The closer the value is to 1, the more accurate it is: <1 means under‐prediction and >1 over‐prediction. If the value is <0.7 or >1.15, it is not a suitable model, that is, it cannot be used and becomes an indicator of whether it is safe or dangerous to use as a growth model (Ross, 1996).

(5) $$B_{f}=10^{\left\{\sum Log(predicted/observed)/n\right\}}$$

A_f_ is an indication of how close observed values obtained from experiments are to predicted values induced from the secondary model formula. A value farther from 1 indicates unfitness of the developed model; however, that does not indicate the safety of the model (Ross, 1996).

(6) $$A_{f}=10^{\left\{\sum |Log(predicted/observed)|/n\right\}}$$

### 2.8 Statistical analysis

All experiments were performed in duplicate and expressed as log colony forming units per gram (log CFU/g), and data from the experiments were used for model development. Statistical analysis was carried out by using the GraphPad Prism 9.0 software (San Diego, California, USA). Statistical significance of growth parameters was analyzed by one‐way analysis of variance (ANOVA) at a significance level of *p* < .05.

## 3 RESULTS AND DISCUSSION

### 3.1 Measurement of pH and water activity of fresh‐cut produce

The pH measurement of fresh‐cut produce used in this study was 6.10 ± 0.13, falling within the range between 6.0 and 7.0, which is optimal for the growth of pathogenic *E*. *coli*. In addition, water activity was 0.987 ± 0.003, close to 0.995, which is the optimal water activity for pathogenic *E*. *coli*. They were extremely suitable conditions for growth of pathogenic *E*. *coli* (MFDS, 2013).

### 3.2 Changes in bacterial cell counts of pathogenic E. coli caused by temperature

**FIGURE 1:**
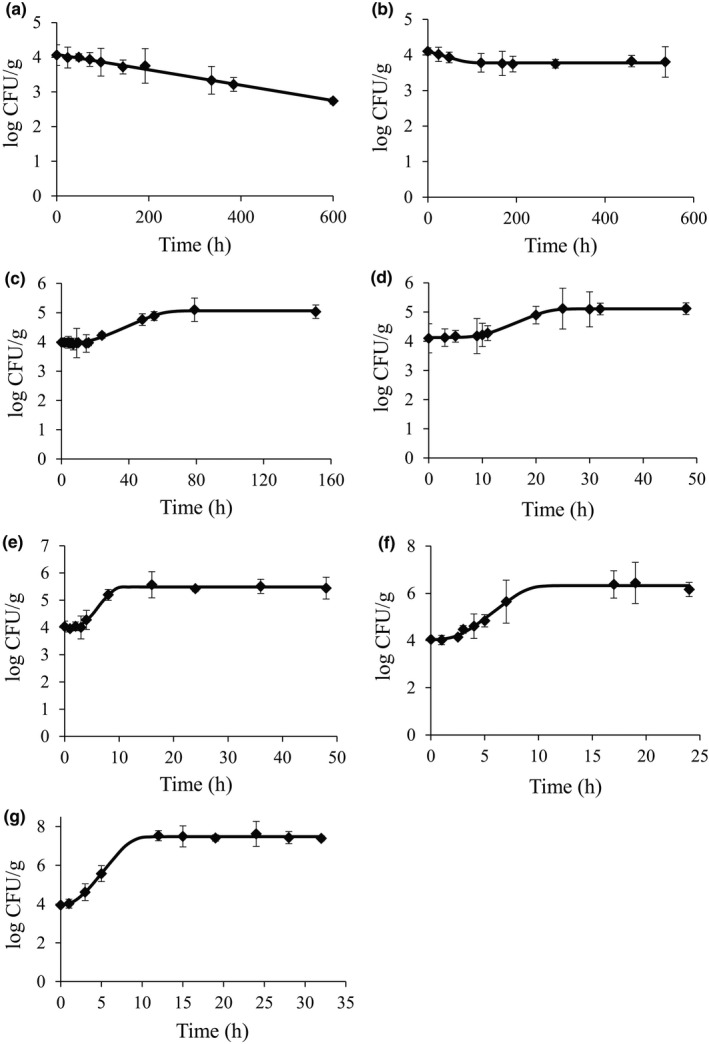
Primary model of the growth of pathogenic *Escherichia coli* in fresh‐cut produce. (a) 4°C, (b) 10°C, (c) 12°C, (d) 15°C, (e) 25°C, (f) 30°C, (g) 37°C

Figure 1 shows the effects of temperature on changes in bacterial cell count of pathogenic *E*. *coli* cocktail. At 4°C and 10°C, a gradual decrease in the number of bacteria was found at the initial concentration of 4 log CFU/g. Fresh‐cut produce stored at 12°C showed little bacterial growth for 17 h; however, the number of bacteria was found to have increased thereafter. These results demonstrated a pattern similar to the growth of *E. coli* O157:H7 in paprika by Yun et al. (2013), where at 10°C, bacteria were maintained at the inoculation levels and subsequently killed, and at 12°C, the number of bacteria gradually increased after 50 h. Other studies showed that *E. coli* O157:H7 started to grow in romaine lettuce at temperatures above 15°C (Khalil & Frank, 2010), and that LPD was observed from 10.5°C in meat (Tamplin et al., 2005). The results of the study above suggest that minimum temperatures for the growth of pathogenic *E*. *coli* vary somewhat depending on the food, thought to be due to the difference in food composition. At 25°C, the LPD was rapidly shortened, resulting in bacterial growth after 3 h. Moreover, approximately 10 h later, its concentration was maintained at 5.4 log CFU/g, an increase of 1.4 log CFU/g compared with the initial concentration. These results demonstrated that growth was rather slow, compared with the reports in which *E. coli* O157:H7 inoculated into paprika showed an increase of 3 log CFU/g after 24 h at 25°C and *E. coli* O157:H7 in romaine lettuce increased by 2.12–2.92 log CFU/g within 24 h (Oliveira et al., 2010). Based on the growth of pathogenic *E*. *coli* at 12, 15, 25, 30, and 37 ℃, it was confirmed that temperature and the rate of growth were proportional. Therefore, for safe ingestion of fresh‐cut produce, it is desirable to effectively control the microbial growth by managing storage time at proper temperature.

### 3.3 Development of the predictive growth model

**FIGURE 2:**
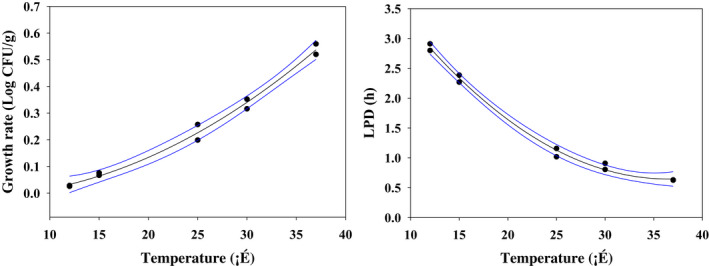
Secondary μ_max_ and LPD models of pathogenic *Escherichia coli* in fresh‐cut produce as a function of temperature (12–37°C). μ_max_, maximum specific growth rate; LPD, lag phase duration

Based on the growth results of pathogenic *E*. *coli* at each temperature (12, 15, 25, 30, and 37 ℃), the μ_max_ and LPD depending on temperatures were calculated using the Baranyi model (Table 1). Overall, μ_max_ was in the range of 0.03–0.54, demonstrating an increase proportionate to temperature, and there was a significant difference at temperatures above 15°C (*p* < .05). In the meanwhile, LPD decreased inversely from 17.38 to 1.87 and significantly decreased at temperatures between 12 and 25°C (*p* < .05). μ_max_ demonstrated a relatively moderate increase according to temperature, while LPD decreased sharply in the range of 15–25°C. *N*_max_, which indicates the final concentration of bacteria, increased as the temperature increased. In addition, to evaluate goodness of fit for the Baranyi model, R^2^ values were analyzed. In this model, the R^2^ values at the five temperatures were within the range of 0.990–0.996, all of which were close to 1, indicating sufficient goodness of fit. Duffy et al. (1994) reported that the R^2^ values of their primary model were within the range of 0.97–0.99, showing sufficient goodness of fit to be used as a parameter for the secondary model. Accordingly, the model developed in this study is considered fit for predicting the growth of pathogenic *E*. *coli*. A secondary model of pathogenic *E*. *coli* was developed according to storage temperatures by applying the polynomial second‐order model to calculated μ_max_ and LPD (Figure 2 and Table 2). μ_max_ and LPD demonstrated superior goodness of fit, having R^2^ values close to 1 (0.990 and 0.994, respectively). Therefore, using the developed model, it is possible to predict the growth of pathogenic *E*. *coli* in fresh‐cut produce according to various storage temperatures, and it is deemed to be fully utilized for risk assessment.

**TABLE 1:** Kinetic parameters calculated by the Baranyi model for the growth of pathogenic *Escherichia coli* in fresh‐cut produce during storage at 12, 15, 25, 30, and 37℃

Temperature (℃)	*μ*_max_	LPD	*N*_0_	*N*_max_	*R* ^2^
12	0.03 ± 0.00^d^	17.38 ± 1.38^a^	3.92 ± 0.04	5.00 ± 0.09	0.991 ± 0.00
15	0.07 ± 0.01^d^	10.26 ± 0.85^b^	4.10 ± 0.03	5.07 ± 0.06	0.990 ± 0.01
25	0.23 ± 0.44^c^	2.98 ± 0.29^c^	3.95 ± 0.04	5.84 ± 0.50	0.992 ± 0.00
30	0.33 ± 0.03^b^	2.36 ± 0.17^c^	4.00 ± 0.05	6.44 ± 0.17	0.991 ± 0.01
37	0.54 ± 0.03^a^	1.87 ± 0.01^c^	3.92 ± 0.03	7.42 ± 0.07	0.996 ± 0.00

Note

Values are mean ± *SD*.

Values in the same column with different superscript letters (a‐d) are significantly different (*p* < .05).

Abbreviations: μ_max_, maximum specific growth rate; LPD, lag phase duration; *N_0_*, initial bacterial cell counts; *N_max_*, final bacterial counts.

**TABLE 2:** Developed secondary model for effect of temperature on LPD and μ_max_ of pathogenic *Escherichia coli* in fresh‐cut produce

Parameter	Equation	R^2^
lnLPD	= 5.517786 – 0.26694 × Temp +0.003656 × Temp^2^	0.994
μ_max_	= −0.01355 – 0.00144 × Temp +0.000441 × Temp^2^	0.990

Abbreviations: μ_max_, maximum specific growth rate; LPD, lag phase duration.

### 3.4 Validation for developed predictive growth model

For verification of the developed model, experiments were conducted at 20°C, a temperature that had not been used during model development; the results were compared with the value predicted from the model developed in this study. RMSE, B_f_, and A_f_ were calculated, and using these values, the goodness of fit of the model was evaluated (Table 3). RMSE and B_f_ values represent the validity of the predictive model and the difference between observed values and predicted values, respectively, with a value closer to 1 being most ideal. Ross (1996) accepted B_f_ values between 0.7 and 1.15 for the predictive pathogen model, where values ranging between 0.9 and 1.05 were considered good. A_f_ values are an indication of how close observed values are to predicted ones. When the two values are identical, the A_f_ value is 1. The farther the value is from 1, the greater the inaccuracy (Ross, 1996). The RMSE of the model developed in this study was 0.084, close to 0, indicating its validity as a predictive model. In addition, the B_f_ value was 0.995, close to 1, which was found to be within the appropriate range (0.9–1.05), and the A_f_ value was 1.011, demonstrating its greater proximity to 1. Therefore, the model developed in this study is considered to have superior goodness of fit and can be used as a predictive growth model for pathogenic *E*. *coli* for fresh‐cut produce.

**TABLE 3:** Validation of secondary model to predict the growth of pathogenic *Escherichia coli* in fresh‐cut produce

Statistic evaluations		
RMSE	B_f_	A_f_
0.084	0.995	1.011

Abbreviations: A_f_, accuracy factor; B_f_, bias factor; RMSE, root mean square error.

## 4 CONCLUSIONS

This study was conducted to ensure the safety of fresh‐cut produce by developing and verifying a predictive growth model for pathogenic *E*. *coli,* which has the potential to contaminate produce. To develop a predictive growth model, *E*. *coli* was inoculated into fresh‐cut produce, which was stored at different temperatures (4, 10, 12, 15, 25, 30, and 37°C), and *E. coli* growth was observed. It was confirmed that growth took place at temperatures above 12°C. This means that any thermal abuse in the fresh‐cut produce processing, distribution, or even at home could give rise to increase in pathogenic *E. coli* in the risk for the consumers. Therefore, for safe ingestion of fresh‐cut produce, it is desirable to effectively control the microbial growth by managing storage time at proper temperature. After drawing a growth curve at temperatures ranging between 12 and 37°C, the primary predictive model was developed with the μ_max_ and LPD values calculated using the Baranyi model. The secondary model was developed as a temperature function for μ_max_ and LPD using polynomial second‐order model. The R^2^ values of μ_max_ and LPD from the secondary model were 0.990 and 0.994, respectively, demonstrating greater goodness of fit as they were close to 1. In addition, RMSE, B_f_, and A_f_ values for verifying goodness of fit of the secondary model showed great proximity to ideal figures, demonstrating that it was suitable as a predictive model. Therefore, the predictive growth model developed in this study can be used for growth prediction of pathogenic *E*. *coli* according to temperature and storage time in industries that manufacture, process, distribute, and market fresh‐cut produce. In addition, the developed model in this study can be applied to quantitative risk assessment of pathogenic *E. coli* in fresh‐cut produce.

## CONFLICT OF INTEREST

The authors declare that they have no conflict of interest.

## AUTHOR CONTRIBUTIONS

**Eungjeong Heo:** Conceptualization (lead); Supervision (lead); Validation (lead); Writing‐review & editing (lead). **You Jin Kim:** Conceptualization (equal); Methodology (lead); Writing‐original draft (lead); Writing‐review & editing (equal). **Ju Yeon Park:** Methodology (equal); Writing‐original draft (supporting). **Soo Hwan Suh:** Writing‐review & editing (equal). **Migyeong Kim:** Supervision (supporting); Validation (supporting). **hyo sun kwak:** Writing‐review & editing (supporting). **Soon‐Han Kim:** Supervision (supporting); Validation (supporting).

## ETHICAL REVIEW

This study does not involve any human or animal testing.

## Data Availability

Due to privacy/ethical restriction, the data that support the findings of this study are available upon reasonable request from the corresponding author.
